# Minimally invasive management versus open surgery in the treatment of penetrating bladder injuries: a retrospective cohort study

**DOI:** 10.1186/s12894-021-00900-w

**Published:** 2021-09-28

**Authors:** John Culhane, Johar Raza Syed, Sameer Siddiqui

**Affiliations:** 1 Department of Trauma, Saint Louis University, 1008 Spring Ave, Saint Louis, MO 63110 USA; 2 Department of Urology, Saint Louis University, 1008 Spring Ave, Saint Louis, MO 63110 USA

**Keywords:** Penetrating, Bladder, Trauma, Minimally invasive

## Abstract

**Background:**

While blunt extra-peritoneal bladder injury is typically treated non-operatively or with minimally invasive management, the treatment for penetrating bladder injury is generally open surgery. We identify a group of patients with penetrating bladder injury who were treated with minimally invasive management and compare the results with those who underwent traditional open surgical treatment.

**Methods:**

This retrospective cohort study analyzes penetrating bladder injuries from a single trauma center from 2012 through 2019, and from the National Trauma Data Bank for 2016 and 2017. Mortality, complications, and length of stay were compared for minimally invasive management versus open surgery. We used Chi square to test significance for categorical variables, Mann–Whitney U test for ordinal variables, and T-test for continuous variables. Multivariate analysis was performed with multiple logistic, ordinal, and linear regression.

**Results:**

Local: 117 (0.63%) had a bladder injury; 30 (25.6%) were penetrating. 6 (20.0%) were successfully treated with minimally invasive management with no complication versus 24 complications in 11 patients (45.8%) for open surgery (*p* = 0.047). Open surgical management was not a significant independent predictor of mortality or hospital length of stay. National Trauma Data Bank: 5330 (0.27%) had a bladder injury; 963 (19.5%) were penetrating. 97 (10.1%) were treated with minimally invasive management. The minimally invasive management group had 12 complications in 5 patients (4.9%) versus 280 complications in 169 patients (19.7%) for open surgery (*p* =  < 0.001). Open surgery was a significant independent predictor of complications (OR 1.57, *p* = 0.003) and longer hospital length of stay (B = 5.31, *p* < 0.001).

**Conclusions:**

Most penetrating bladder injury requires open surgery, however a small proportion can safely be managed with minimally invasive management. Minimally invasive management is associated with lower total complications and shorter hospital length of stay in select patients.

## Introduction

Selective non-operative or minimally invasive management (MIM) has become standard of care for blunt extraperitoneal bladder injury, however MIM for penetrating bladder injury remains controversial. MIM can reduce the morbidity of large open incisions and potentially decrease recovery time, but this must be balanced against the risk of delayed diagnosis and treatment which could lead to peritonitis, abscess, and other complications. There is disagreement among major guidelines regarding penetrating mechanism as an absolute indication for open exploration and repair.

The latest American Association for the Surgery of Trauma (AAST) urologic trauma guidelines recommend selective MIM of extraperitoneal bladder injury for both blunt and penetrating trauma [1]. European Urologic Association (EUA) guidelines recommend operative treatment of all penetrating bladder injury [2], and American Urologic Association (AUA) guidelines do not distinguish treatment based on mechanism [3]. The individual studies that form the basis of the guidelines include very little penetrating trauma. Often the mechanism is not specified, or blunt and penetrating cases are grouped together. We present a retrospective cohort study of exclusively penetrating injuries in which MIM was implemented. Our local study is supplemented by a review of the National Trauma Data Bank (NTDB). Our hypothesis is that MIM can be performed safely for a carefully selected subset of penetrating bladder injuries. We believe that ours is the first study focused specifically on MIM of penetrating bladder injury.

## Materials and methods

### Study design and population

This is a retrospective cohort study that compares outcomes of patients who underwent open surgical versus minimally invasive management of penetrating bladder trauma. Local patients were treated at a level I trauma center with a prospectively maintained trauma database. After internal review board approval, penetrating bladder injuries between 2012 and 2019 were queried. We defined MIM as observation, prolonged bladder drainage, or use of endoscopic procedures such as cystoscopy. We excluded both open and laparoscopic bladder repair. Any patient who was coded for open peritoneal inspection or any other procedure that is normally done via an open trans-peritoneal approach was placed into the open group.

Patients were chosen for MIM based on hemodynamic stability, lack of peritonitis, and no intraperitoneal contrast extravasation on CT cystogram. Patients with other indications for open pelvic surgery such as orthopedic procedures, vascular repair, and repair of full thickness rectal perforation would also undergo open bladder repair.

The NTDB is a registry of trauma data from multiple US trauma centers. The years 2016 and 2017 were chosen because these are the first years which use ICD10 coding, which is necessary to classify diagnoses and procedures with adequate precision for this study. NTDB and local patients were analyzed separately rather than pooling the cases because differences in reporting could introduce bias, and because access to the charts provides more detail for the local cohort.

### Data collection and statistical analysis

Chi square was used to test for significance for categorical variables. Mann–Whitney U test was used to test for significance for ordinal variables. T-test and Mann–Whitney U test were used to test for significance for continuous variables based on the sample size and distribution. Multivariate analysis was performed with multiple logistic regression for binary categorical outcome, multiple ordinal regression for ordinal outcome, and multiple linear regression for continuous outcome. The independent variables for the regressions are open surgery, age, gender, race, comorbidity index, trauma injury severity score (TRISS), university versus non-university hospital, verification level of trauma center, firearm injury, and grade of bladder injury according to the AAST Trauma Organ Injury Scale (OIS).

A comorbidity index was created by assigning a point to major comorbid conditions and totaling them into a single numerical score. We compared patients who had no open surgery (minimally invasive management—MIM), with those who had an open urologic procedure or other open peritoneal surgery. We chose these groups to examine whether it was safe to omit open exploration for both diagnosis and treatment. The MIM group would be most likely to suffer the consequences of missed or undertreated injury, and to show the benefits of less invasive treatment.

All statistical analysis was conducted with SPSS Statistics, version 26.0 (IBM Corp., Armonk, N.Y., USA).

## Results

### Patient characteristics

There were 18,506 trauma patients treated at our level I trauma center during the years 2012 through 2019. 1,961,104 NTDB patients were analyzed for the years 2016 and 2017. For the local group, 117 (0.63%) had a bladder injury. 30 (25.6%) of the injuries were penetrating. Discharge disposition is missing for one patient. The other 29 all survived to discharge. The injury was due to a firearm for all patients. For the NTDB group, 5330 (0.27%) had a bladder injury. 1042 (19.5%) of the injuries were penetrating. We eliminated 71 patients who died or were transferred within 24 h because they may not have undergone bladder surgery due to short survival or hospital stay. This leaves 963 to analyze. The mechanisms of injury were: 911 (94.7%) firearms, 48 (5.0%) sharp objects, 3 (0.3%) lawn mower, 1 (0.1%) nail gun. Baseline characteristics for bladder injury patients are reported in Table 1.

**Table 1:** Baseline characteristics of penetrating bladder injury patients

Characteristics	MIM	Open
*Local data*		
Sex—Female	3 (50.0%)	2 (8.3%)
Race—White	2 (33.3%)	4 (16.7%)
Chest Injury AIS > 2	0 (0.0%)	2 (8.3%)
Abdominal Injury AIS > 2	0 (0.0%)	14(58.3%)
Extremity Injury AIS > 2	4 (66.7%)	17(70.8%)
Rectal Injury	0(0%)	5(16.7%)
Pelvic Fracture	1(16.7%)	5(16.7%)
Abdomino-Pelvic Vascular Injury	0(0%)	7(23.3%)
Age	33.3	31
BMI	28.4	27.5

	MIM		Open	
	Mean	Median	Mean	Median
Injury severity Score	11.2	13	17.5	18
TRISS	0.98		0.95	
Comorbidity Index	0.67	1	1.4	1

Characteristics	MIM	Open
*NTDB data*		
Sex—Female	12 11.7%)	74 (8.6%)
Race—White	37(35.9%)	227(26.4%)
Head Injury AIS > 2	1 (1.0%)	12 (1.4%)
Chest Injury AIS > 2	4 (3.9%)	71 (8.3%)
Abdominal Injury AIS > 2	33(32.0%)	602(70.0%)
Extremity Injury AIS > 2	37(35.9%)	308(35.8%)
Rectal Injury	6(6.2%)	216(24.9%)
Pelvic Fracture	47(48.4%)	368(42.5%)
Abdomino-Pelvic vascular injury	6(6.2%)	114(13.2%)
University Hospital	61(59.2%)	516(60.0%)
Level 1 Designation	46(44.7%)	420(48.8%)
Firearm Injury	90(87.4%)	821(95.5%)
Age	32	29.9
BMI	27.5	27.1

	MIM		Open	
	Mean	Median	Mean	Median
Injury severity Score	12.1	12	15.5	14
TRISS	0.97		0.94	
Comorbidity index	0.19	0	0.21	0

### Type of surgery

For the local patients, 6 (20.0%) were treated without an open bladder repair or inspection. All injuries were extraperitoneal. Three were AAST grade I, consisting of 3 bladder hematomas, 2 of which were associated with proximal urethral injuries which were treated endoscopically. Three were grade II, consisting of extraperitoneal injuries with contained extravasation of contrast. All were treated with drainage only. None failed non-operative management.

For the NTDB, 866 (89.9%) patients had surgical bladder repair or other open peritoneal surgery. Presumably, the bladder was inspected during open surgery, although it is not always recorded. 97 (10.1%) did not have any open urologic procedure, surgical bladder repair or open peritoneal surgery. This comprises the MIM group. Mechanisms of injury in this group consist of 89 GSW, 10 sharp objects, 1 nail gun, and 2 lawn mowers.

Of the 97 MIM patients, 43 had 65 minimally invasive urologic procedures, 32 of which were drainage. 75 (7.8%) of penetrating bladder injury patients did not have open peritoneal surgery or any urologic procedure other than drainage. Urologic injury in this group was treated with drainage and observation only.

The NTDB recorded the location of the injury for 54 of the patients. 51 of these were classified as extraperitoneal. Exceptions were 2 patients who had an organ injury scale Grade IV injury. A 27 year old male had laparoscopic inspection and drainage of the peritoneal cavity but no repair. An 18 y/o male went to the floor from the ED. He was ultimately discharged to court/law enforcement. A fourteen year old male had a Grade V injury. He had laparoscopic inspection of the peritoneal cavity and drainage of the bladder, but no repair. He survived to discharge home.

### Outcomes and complications

#### Univariate analysis

The local MIM group had no complications versus 24 complications in 11 patients (45.8%) for open surgery: *p* = 0.047. Average length of stay (LOS) was 5.5 days for MIM versus 15.1 days for open surgery (*p* = 0.03). The NTDB MIM group had 12 complications in 5 patients (4.9%) versus 280 complications in 169 patients (19.7%) for open surgery (*p* =  < 0.001). Average LOS was 6.1 days for MIM versus 12.9 days for open surgery (*p* = 0.01). This represents significantly decreased overall complications and average LOS with MIM for both the local and NTDB groups. Intensive Care Unit (ICU) LOS was not significantly different for either group.

#### Multivariate analysis

For the local group multiple linear regression showed that open surgery was a significant predictor of increased LOS: B = 9.72 (1.10 to 18.33) *p* =  < 0.029, meaning that open surgery predicted a 9.72 day average increase in LOS. Other independent predictors were TRISS and severe chest injury. Open surgery was not an independent predictor of ICU LOS or complications. Since no local patients died, multivariate analysis was not conducted for mortality.

For the NTDB group, multiple logistic regression showed that age and TRISS, but not open surgery were significant independent predictors of mortality. Multivariate ordinal regression showed that open surgery was associated with 1.57 (0.544 to 2.60) unit increase in the log odds of being in a group with a higher number of complications (*p* = 0.003). Other independent predictors of increased complications include age, TRISS, and number of comorbidities. Multivariate linear regression showed that open surgery predicted a 5.31 day increase in LOS (2.45 to 8.16, *p* < 0.001). Open surgery did not predict increased ICU LOS.

## Discussion

MIM has become standard for many blunt injuries, but acceptance has been slower for penetrating trauma. As with trauma in general, there has been a shift to MIM for urologic trauma, including bladder injuries. Not all bladder injuries are appropriate for MIM. Major guidelines agree that surgeons should repair all intraperitoneal bladder injuries, both blunt and penetrating [2–6]. Hemodynamically unstable patients and patients with other hollow viscus injuries are not candidates for MIM [6]. For extraperitoneal injury, operative repair is recommended if the patient is already undergoing pelvic surgery for another injury such as exploratory laparotomy or orthopedic repair of the pelvis [2, 3]. Complicated injuries involving the ureteral orifice or trigone may require surgery to prevent future incontinence [4].

These restrictions leave hemodynamically stable patients with extraperitoneal injuries and no other indication for pelvic surgery as potential candidates for MIM. Most studies of MIM of bladder injury are in the blunt setting, where it has become the standard of care, however there is disagreement among urologic trauma guidelines regarding whether the blunt bladder injury paradigm applies to penetrating bladder injury (PBI) as well. Some advocate a mandatory surgical approach. A 2004 consensus statement by Gomez et al. and the 2005 European Urologic Association (EUA) guidelines recommend surgical exploration of all PBI [7, 8]. In a 2015 review of lower urinary tract injuries by the EUA trauma guidelines panel, Lumen et al. strongly recommended that all PBI should undergo emergency exploration with bladder repair [2]. Stein published a narrative review and update on 2014 AUA guidelines based on personal experience. The author’s conclusion was that penetrating injuries should be explored and treated primarily [9]. Phillips published a review in 2017 which listed penetrating mechanism as an indication for open surgery, citing the AUA university core curriculum [10].

In contrast, in a 2019 statement, AAST urologic trauma guidelines recommend MIM of extraperitoneal bladder injury for both blunt and penetrating trauma [1]. Other guidelines do not explicitly state whether the recommendation applies to both blunt and penetrating injury. In the 2004 Eastern Association trauma guidelines for genitourinary trauma for all mechanisms, Holevar reported that catheter drainage shows results equal to surgical repair [6]. The latest AUA guidelines recommend MIM with drainage for extraperitoneal bladder injury without mention of injury mechanism [3]. Bryk published a 2013 review of urologic trauma guidelines. The recommendation after synthesizing all guidelines was that MIM of extraperitoneal injuries is acceptable without specifying the mechanism of injury [11].

Evidence for MIM of bladder injury consists mainly of retrospective reviews which contain few cases of penetrating injury. In some studies, the mechanism is not specified. If there are any penetrating injuries, they are grouped together with blunt [12–16]. There are few examples in the literature focused on PBI. Zaid et al. published a narrative review of penetrating urologic injury. The authors stated that PBI can be managed non-operatively, but the reference provided [7], another review, states that operative exploration and repair is the standard approach [17].

## Limitations

As with any large multi-center database, the NTDB has inherent limitations with respect to granularity and coding accuracy [18]. The choice of MIM tends to select patients with less severe injuries, which could account for the shorter LOS and lower complication rate in the univariate analysis. We included the TRISS and AAST grade of bladder injury in the multivariate analyses to adjust for severity of injury, but subtle biases could remain. All injuries managed with MIM in the local group and the vast majority of those with location recorded in the NTDB group were extraperitoneal. We believe that the most conservative policy is to restrict MIM to extraperitoneal injuries. Our population includes mainly firearm lesions. Results could differ for populations with different injury patterns.

In summary, reports of MIM of PBI in the literature are rare, making it difficult to generate consistent treatment guidelines. For the small number of cases reported, the practice appears safe. Our report adds 6 cases from our institution and 97 cases from the NTDB to the total. All local patients were managed successfully without complication. Total complications were significantly lower for both local and NTDB cohorts and LOS was lower for the NTDB cohort, thus in our study, the practice appears safe as well.

## Conclusion

Minimally invasive management of penetrating bladder injury remains controversial. Our analysis describes successful minimally invasive management of PBI, but it is important to bear in mind that this only applies to a narrow subset of patients. We propose the following criteria for MIM of PBI: hemodynamically stable patients with no other indication for pelvic surgery, extraperitoneal location, and partial thickness injury or well contained extraperitoneal contrast extravasation (AAST Grades I and II). Our results are largely based on firearm lesions, therefore outcome may differ for other mechanisms.

Patients meeting these criteria may be managed without any open exploration or any other open urologic procedure. MIM of PBI may involve minimally invasive percutaneous or endoscopic techniques, but some injuries may be managed with observation only, omitting urologic procedures of any kind. MIM of PBI is not associated with increased mortality or increase of any specific reported complication. MIM is associated with significantly fewer total complications and is an independent predictor of decreased hospital LOS. We believe that these results indicate that for a minority of PBI patients, observation and if necessary, endoscopic urologic procedures represent a safe treatment option.

## Data Availability

The NTDB is publicly available from the American College of Surgeons. Local institutional patient data is considered confidential. To request data, please contact the corresponding author, John Culhane, at john.culhane@health.slu.edu.

## Abbreviations

MIM: Minimally invasive management
NTDB: National trauma data bank
AAST: American association for the surgery of trauma
OIS: Organ injury scale
AUA: American Urologic Association
EUA: European Urologic Association
ICU: Intensive care unit
LOS: Length of stay
ISS: Injury severity score
TRISS: Trauma injury severity score
GSW: Gunshot Wound
